# 1-(1,3-Benzodioxol-5-yl)pentan-1-one

**DOI:** 10.1107/S1600536808040257

**Published:** 2008-12-20

**Authors:** Xiao-Ming Wang, Wei Cheng, Yu-Wang Han

**Affiliations:** a Department of Applied Chemistry, College of Science, Nanjing University of Technology, Nanjing 210009, People’s Republic of China

## Abstract

In the mol­ecule of title compound, C_12_H_14_O_3_, the benzodioxole ring system is essentially planar. In the crystal structure, weak inter­molecular C—H⋯O hydrogen bonds link mol­ecules into chains along the *c* axis, and π–π contacts between dioxole rings and between dioxole and benzene rings of the benzodioxole ring systems [centroid–centroid distances 3.702 (3) and 3.903 (3) Å] may further stabilize the structure. Two C—H⋯π inter­actions are also found.

## Related literature

For general background, see: Koeppe *et al.* (1969 ▶). For a related structure, see: May *et al.* (2000 ▶). For bond-length data, see: Allen *et al.* (1987 ▶);
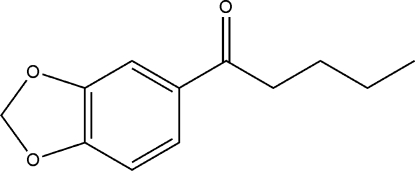


## Experimental

### 

#### Crystal data

C_12_H_14_O_3_
*M* *_r_* = 206.23
Monoclinic, 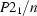

*a* = 6.7940 (14) Å
*b* = 12.960 (3) Å
*c* = 12.244 (2) Å
β = 93.46 (3)°
*V* = 1076.1 (4) Å^3^
*Z* = 4
Mo *K*α radiation
μ = 0.09 mm^−1^
*T* = 298 (2) K
0.30 × 0.20 × 0.10 mm

#### Data collection

Enraf–Nonius CAD-4 diffractometer
Absorption correction: ψ scan (North *et al.*, 1968 ▶) *T*_min_ = 0.973, *T*_max_ = 0.991
2133 measured reflections
1961 independent reflections
1079 reflections with *I* > 2σ(*I*)
*R*_int_ = 0.031
3 standard reflections frequency: 120 min intensity decay: 1%

#### Refinement

*R*[*F* ^2^ > 2σ(*F* ^2^)] = 0.068
*wR*(*F* ^2^) = 0.171
*S* = 1.00
1961 reflections
136 parameters
H-atom parameters constrained
Δρ_max_ = 0.19 e Å^−3^
Δρ_min_ = −0.20 e Å^−3^

### 

Data collection: *CAD-4 Software* (Enraf–Nonius, 1985 ▶); cell refinement: *CAD-4 Software*; data reduction: *XCAD4* (Harms & Wocadlo, 1995 ▶); program(s) used to solve structure: *SHELXS97* (Sheldrick, 2008 ▶); program(s) used to refine structure: *SHELXL97* (Sheldrick, 2008 ▶); molecular graphics: *PLATON* (Spek, 2003 ▶); software used to prepare material for publication: *SHELXTL* (Sheldrick, 2008 ▶) and *PLATON*.

## Supplementary Material

Crystal structure: contains datablocks I, global. DOI: 10.1107/S1600536808040257/hk2587sup1.cif

Structure factors: contains datablocks I. DOI: 10.1107/S1600536808040257/hk2587Isup2.hkl

Additional supplementary materials:  crystallographic information; 3D view; checkCIF report

## Figures and Tables

**Table 1:** Hydrogen-bond geometry (Å, °)

*D*—H⋯*A*	*D*—H	H⋯*A*	*D*⋯*A*	*D*—H⋯*A*
C8—H8*A*⋯O1^i^	0.93	2.60	3.419 (4)	148
C3—H3*A*⋯*Cg*2^ii^	0.97	2.99	3.831 (3)	145
C12—H12*A*⋯*Cg*2^iii^	0.97	2.84	3.633 (3)	139

Symmetry codes: (i) 
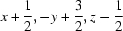
; (ii) 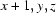; (iii) 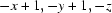. *Cg*2 is the centroid of the C6–C11 ring.

**Fig. 1.**
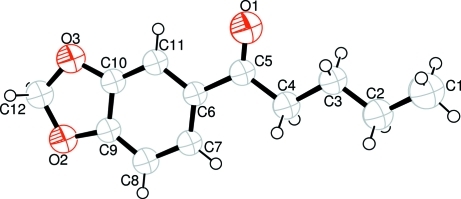
The molecular structure of the title molecule, with the atom-numbering scheme.

**Fig. 2.**
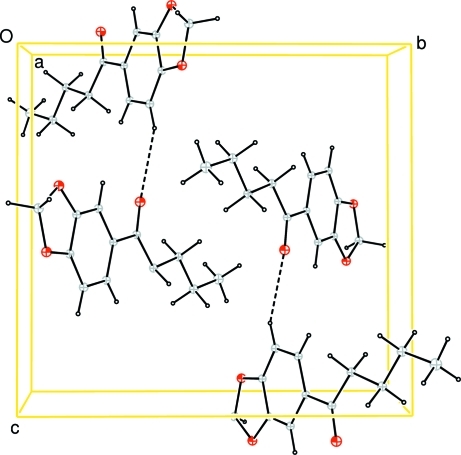
A partial packing diagram of the title compound. Hydrogen bonds are shown as dashed lines.

## supplementary crystallographic information

### Comment

The title compound is an important medicine intermediate used to synthesize methylenedioxypyrovalerone (Koeppe *et al.*,  1969). As part of our studies in this area, we report herein its crystal structure.

In the molecule of title compound (Fig 1), the bond lengths (Allen *et al.*,  1987) and angles are within normal ranges. Rings A (O2/O3/C9/C10/C12) and B (C6-C11) are, of course, planar, and the dihedral angle between them is A/B = 0.56 (3)°. So, they are also coplanar. Atoms O1, C5 and C4 are 0.011 (3), -0.049 (3) and -0.119 (3) Å away from the plane of the benzodioxole ring system.

In the crystal structure, weak intermolecular C-H···O hydrogen bonds (Table 1) link the molecules into chains along the c axis (Fig. 2), in which they may be effective in the stabilization of the structure. The π-π contacts between the dioxole rings and the dioxole and benzene rings of the benzodioxole ring systems, Cg1—Cg1^i^ and Cg1—Cg2^i^ [symmetry code: (i) 1 - x, 1 - y, -z where Cg1 and Cg2 are centroids of the rings A (O2/O3/C9/C10/C12) and B (C6-C11), respectively] may further stabilize the structure, with centroid-centroid distances of 3.702 (3) Å and 3.903 (3) Å. There also exist two C–H···π interactions (Table 1).

### Experimental

The title compound was synthesized according to a literature method (May *et al.*,  2000). Crystals suitable for X-ray analysis were obtained by dissolving the title compound (0.2 g) in methanol (25 ml), and evaporating the solvent slowly at room temperature for about 7 d.

### Refinement

H atoms were positioned geometrically, with C-H = 0.93, 0.97 and 0.96 Å for aromatic, methylene and methyl H, respectively, and constrained to ride on their parent atoms with U_iso_(H) = xU_eq_(C), where x = 1.5 for methyl H and x = 1.2 for all other H atoms.

### Crystal data

**Table:**

C_12_H_14_O_3_	*F*(000) = 440
*M**_r_* = 206.23	*D*_x_ = 1.273 Mg m^−^^3^
Monoclinic, *P*2_1_/*n*	Mo *K*α radiation, λ = 0.71073 Å
Hall symbol: -P 2yn	Cell parameters from 25 reflections
*a* = 6.7940 (14) Å	θ = 9–12°
*b* = 12.960 (3) Å	µ = 0.09 mm^−^^1^
*c* = 12.244 (2) Å	*T* = 298 K
β = 93.46 (3)°	Needle, colorless
*V* = 1076.1 (4) Å^3^	0.30 × 0.20 × 0.10 mm
*Z* = 4	

### Data collection

**Table:**

Enraf–Nonius CAD-4 diffractometer	1079 reflections with *I* > 2σ(*I*)
Radiation source: fine-focus sealed tube	*R*_int_ = 0.031
graphite	θ_max_ = 25.3°, θ_min_ = 2.3°
ω/2θ scans	*h* = 0→8
Absorption correction: ψ scan (North *et al.*, 1968)	*k* = 0→15
*T*_min_ = 0.973, *T*_max_ = 0.991	*l* = −14→14
2133 measured reflections	3 standard reflections every 120 min
1961 independent reflections	intensity decay: 1%

### Refinement

**Table:**

Refinement on *F*^2^	Primary atom site location: structure-invariant direct methods
Least-squares matrix: full	Secondary atom site location: difference Fourier map
*R*[*F*^2^ > 2σ(*F*^2^)] = 0.068	Hydrogen site location: inferred from neighbouring sites
*wR*(*F*^2^) = 0.171	H-atom parameters constrained
*S* = 1.00	*w* = 1/[σ^2^(*F*_o_^2^) + (0.06*P*)^2^ + 0.6*P*] where *P* = (*F*_o_^2^ + 2*F*_c_^2^)/3
1961 reflections	(Δ/σ)_max_ < 0.001
136 parameters	Δρ_max_ = 0.19 e Å^−^^3^
0 restraints	Δρ_min_ = −0.20 e Å^−^^3^

### Special details

**Table:**

Geometry. All e.s.d.'s (except the e.s.d. in the dihedral angle between two l.s. planes) are estimated using the full covariance matrix. The cell e.s.d.'s are taken into account individually in the estimation of e.s.d.'s in distances, angles and torsion angles; correlations between e.s.d.'s in cell parameters are only used when they are defined by crystal symmetry. An approximate (isotropic) treatment of cell e.s.d.'s is used for estimating e.s.d.'s involving l.s. planes.
Refinement. Refinement of *F*^2^ against ALL reflections. The weighted *R*-factor *wR* and goodness of fit *S* are based on *F*^2^, conventional *R*-factors *R* are based on *F*, with *F* set to zero for negative *F*^2^. The threshold expression of *F*^2^ > σ(*F*^2^) is used only for calculating *R*-factors(gt) *etc*. and is not relevant to the choice of reflections for refinement. *R*-factors based on *F*^2^ are statistically about twice as large as those based on *F*, and *R*- factors based on ALL data will be even larger.

### Fractional atomic coordinates and isotropic or equivalent isotropic displacement parameters (Å^2^)

**Table:**

	*x*	*y*	*z*	*U*_iso_*/*U*_eq_	
O1	0.0098 (4)	0.8069 (2)	1.06817 (19)	0.0789 (9)	
O2	0.7749 (3)	0.5827 (2)	0.94774 (18)	0.0650 (7)	
O3	0.6590 (4)	0.6096 (2)	1.11889 (18)	0.0666 (8)	
C1	−0.4973 (6)	1.0228 (4)	0.8325 (3)	0.0911 (14)	
H1A	−0.5608	1.0466	0.7650	0.137*	
H1B	−0.4467	1.0809	0.8741	0.137*	
H1C	−0.5909	0.9864	0.8738	0.137*	
C2	−0.3317 (5)	0.9524 (3)	0.8088 (3)	0.0678 (11)	
H2A	−0.2416	0.9894	0.7644	0.081*	
H2B	−0.3849	0.8951	0.7655	0.081*	
C3	−0.2171 (5)	0.9099 (3)	0.9067 (3)	0.0589 (9)	
H3A	−0.3054	0.8699	0.9495	0.071*	
H3B	−0.1680	0.9670	0.9519	0.071*	
C4	−0.0454 (5)	0.8428 (3)	0.8797 (3)	0.0567 (9)	
H4A	−0.0947	0.7866	0.8333	0.068*	
H4B	0.0435	0.8833	0.8379	0.068*	
C5	0.0698 (5)	0.7978 (3)	0.9768 (3)	0.0519 (9)	
C6	0.2532 (5)	0.7393 (2)	0.9607 (2)	0.0458 (8)	
C7	0.3230 (5)	0.7220 (3)	0.8582 (2)	0.0507 (9)	
H7A	0.2519	0.7472	0.7966	0.061*	
C8	0.4974 (5)	0.6678 (3)	0.8456 (3)	0.0566 (9)	
H8A	0.5421	0.6546	0.7766	0.068*	
C9	0.5996 (5)	0.6350 (3)	0.9378 (3)	0.0501 (8)	
C10	0.5302 (5)	0.6509 (3)	1.0409 (2)	0.0503 (8)	
C11	0.3604 (5)	0.7021 (3)	1.0545 (2)	0.0514 (9)	
H11A	0.3155	0.7125	1.1239	0.062*	
C12	0.8125 (5)	0.5645 (3)	1.0614 (3)	0.0627 (10)	
H12A	0.8183	0.4908	1.0754	0.075*	
H12B	0.9382	0.5945	1.0860	0.075*	

### Atomic displacement parameters (Å^2^)

**Table:**

	*U*^11^	*U*^22^	*U*^33^	*U*^12^	*U*^13^	*U*^23^
O1	0.0840 (19)	0.098 (2)	0.0557 (15)	0.0266 (17)	0.0117 (13)	0.0023 (14)
O2	0.0596 (16)	0.0744 (18)	0.0610 (15)	0.0185 (14)	0.0035 (12)	−0.0015 (13)
O3	0.0670 (17)	0.0770 (18)	0.0552 (14)	0.0171 (14)	−0.0010 (12)	0.0033 (13)
C1	0.101 (3)	0.085 (3)	0.085 (3)	0.032 (3)	−0.015 (3)	−0.012 (2)
C2	0.074 (3)	0.064 (2)	0.065 (2)	0.014 (2)	0.0040 (19)	0.0096 (19)
C3	0.063 (2)	0.046 (2)	0.067 (2)	−0.0010 (18)	0.0010 (18)	−0.0020 (18)
C4	0.055 (2)	0.058 (2)	0.0580 (19)	−0.0002 (18)	0.0049 (16)	0.0037 (18)
C5	0.059 (2)	0.048 (2)	0.0497 (18)	−0.0008 (17)	0.0083 (16)	−0.0009 (16)
C6	0.049 (2)	0.0390 (18)	0.0498 (17)	−0.0036 (16)	0.0070 (15)	0.0020 (14)
C7	0.055 (2)	0.050 (2)	0.0460 (17)	−0.0029 (18)	−0.0016 (15)	0.0032 (15)
C8	0.063 (2)	0.060 (2)	0.0479 (18)	−0.0032 (19)	0.0119 (17)	−0.0051 (16)
C9	0.054 (2)	0.044 (2)	0.0521 (18)	−0.0044 (17)	0.0040 (16)	−0.0030 (15)
C10	0.059 (2)	0.044 (2)	0.0469 (17)	−0.0014 (17)	−0.0016 (16)	0.0008 (15)
C11	0.057 (2)	0.053 (2)	0.0443 (17)	0.0006 (18)	0.0054 (16)	−0.0015 (15)
C12	0.062 (2)	0.061 (2)	0.064 (2)	0.006 (2)	0.0000 (18)	0.0036 (19)

### Geometric parameters (Å, °)

**Table:**

O1—C5	1.220 (4)	C4—C5	1.501 (4)
O2—C9	1.370 (4)	C4—H4A	0.9700
O2—C12	1.419 (4)	C4—H4B	0.9700
O3—C10	1.365 (4)	C5—C6	1.482 (4)
O3—C12	1.419 (4)	C6—C7	1.387 (4)
C1—C2	1.491 (5)	C6—C11	1.407 (4)
C1—H1A	0.9600	C7—C8	1.394 (5)
C1—H1B	0.9600	C7—H7A	0.9300
C1—H1C	0.9600	C8—C9	1.358 (5)
C2—C3	1.495 (4)	C8—H8A	0.9300
C2—H2A	0.9700	C9—C10	1.389 (4)
C2—H2B	0.9700	C10—C11	1.349 (4)
C3—C4	1.508 (4)	C11—H11A	0.9300
C3—H3A	0.9700	C12—H12A	0.9700
C3—H3B	0.9700	C12—H12B	0.9700
			
C9—O2—C12	105.9 (3)	O1—C5—C4	120.1 (3)
C10—O3—C12	105.9 (2)	C6—C5—C4	119.8 (3)
C2—C1—H1A	109.5	C7—C6—C11	119.6 (3)
C2—C1—H1B	109.5	C7—C6—C5	122.6 (3)
H1A—C1—H1B	109.5	C11—C6—C5	117.8 (3)
C2—C1—H1C	109.5	C6—C7—C8	121.4 (3)
H1A—C1—H1C	109.5	C6—C7—H7A	119.3
H1B—C1—H1C	109.5	C8—C7—H7A	119.3
C1—C2—C3	115.6 (3)	C9—C8—C7	117.4 (3)
C1—C2—H2A	108.4	C9—C8—H8A	121.3
C3—C2—H2A	108.4	C7—C8—H8A	121.3
C1—C2—H2B	108.4	C8—C9—O2	128.8 (3)
C3—C2—H2B	108.4	C8—C9—C10	121.7 (3)
H2A—C2—H2B	107.5	O2—C9—C10	109.5 (3)
C2—C3—C4	114.1 (3)	C11—C10—O3	128.6 (3)
C2—C3—H3A	108.7	C11—C10—C9	121.6 (3)
C4—C3—H3A	108.7	O3—C10—C9	109.8 (3)
C2—C3—H3B	108.7	C10—C11—C6	118.2 (3)
C4—C3—H3B	108.7	C10—C11—H11A	120.9
H3A—C3—H3B	107.6	C6—C11—H11A	120.9
C5—C4—C3	115.1 (3)	O3—C12—O2	108.9 (3)
C5—C4—H4A	108.5	O3—C12—H12A	109.9
C3—C4—H4A	108.5	O2—C12—H12A	109.9
C5—C4—H4B	108.5	O3—C12—H12B	109.9
C3—C4—H4B	108.5	O2—C12—H12B	109.9
H4A—C4—H4B	107.5	H12A—C12—H12B	108.3
O1—C5—C6	120.1 (3)		
			
C1—C2—C3—C4	−177.5 (3)	C12—O2—C9—C10	0.9 (4)
C2—C3—C4—C5	−179.0 (3)	C12—O3—C10—C11	180.0 (4)
C3—C4—C5—O1	8.2 (5)	C12—O3—C10—C9	−1.2 (4)
C3—C4—C5—C6	−173.9 (3)	C8—C9—C10—C11	−1.8 (5)
O1—C5—C6—C7	176.7 (3)	O2—C9—C10—C11	179.1 (3)
C4—C5—C6—C7	−1.3 (5)	C8—C9—C10—O3	179.3 (3)
O1—C5—C6—C11	−4.6 (5)	O2—C9—C10—O3	0.2 (4)
C4—C5—C6—C11	177.5 (3)	O3—C10—C11—C6	179.0 (3)
C11—C6—C7—C8	0.4 (5)	C9—C10—C11—C6	0.2 (5)
C5—C6—C7—C8	179.1 (3)	C7—C6—C11—C10	0.4 (5)
C6—C7—C8—C9	−1.8 (5)	C5—C6—C11—C10	−178.4 (3)
C7—C8—C9—O2	−178.6 (3)	C10—O3—C12—O2	1.8 (4)
C7—C8—C9—C10	2.5 (5)	C9—O2—C12—O3	−1.7 (4)
C12—O2—C9—C8	−178.1 (4)		

### Hydrogen-bond geometry (Å, °)

**Table:**

*D*—H···*A*	*D*—H	H···*A*	*D*···*A*	*D*—H···*A*
C8—H8A···O1^i^	0.93	2.60	3.419 (4)	148
C3—H3A···Cg2^ii^	0.97	2.99	3.831 (3)	145
C12—H12A···Cg2^iii^	0.97	2.84	3.633 (3)	139

Symmetry codes: (i) *x*+1/2, −*y*+3/2, *z*−1/2; (ii) *x*+1, *y*, *z*; (iii) −*x*+1, −*y*+1, −*z*.

## References

[bb1] Allen, F. H., Kennard, O., Watson, D. G., Brammer, L., Orpen, A. G. & Taylor, R. (1987). *J. Chem. Soc. Perkin Trans. 2*, pp. S1–19.

[bb2] Enraf–Nonius (1985). *CAD-4 Software* Enraf–Nonius, Delft, The Netherlands.

[bb3] Harms, K. & Wocadlo, S. (1995). *XCAD4* University of Marburg, Germany.

[bb4] Koeppe, H., Ludwig, G. & Zeile, K. (1969). Boehringer Ingelheim GmbH, US Patent No. 3478050.

[bb5] May, P. J., Bradley, M., Harrowven, D. C. & Pallin, D. (2000). *Tetrahedron Lett.* **41**, 1627–1630.

[bb6] North, A. C. T., Phillips, D. C. & Mathews, F. S. (1968). *Acta Cryst.* A**24**, 351–359.

[bb7] Sheldrick, G. M. (2008). *Acta Cryst.* A**64**, 112–122. 10.1107/S0108767307043930 18156677

[bb8] Spek, A. L. (2003). *J. Appl. Cryst.* **36**, 7–13.

